# Coproduced assessments of climate change adaptation reveal equity challenges in locally led approaches

**DOI:** 10.1088/1748-9326/ae63e5

**Published:** 2026-05-19

**Authors:** Ben C Howard, Cynthia Awuni, Samuel Agyei-Mensah, Camilla Audia, Frans Berkhout, Lee D Bryant, Alicia Cavanaugh, Alex Curran, Shona Macleod, Robert Manteaw, Paul Mitchell, Annie Ockelford, Victoria Pratt, Abubakar Sadiq Mohammed, Jacob Tetteh, Wouter Buytaert

**Affiliations:** 1 Department of Civil and Environmental Engineering, Imperial College London, London, United Kingdom; 2 Department of Hospitality and Tourism Management, Tamale Technical University, Tamale, Ghana; 3 Department of Geography and Resource Management, University of Ghana, Accra, Ghana; 4 Institute for Global Sustainable Development, University of Warwick, Coventry, United Kingdom; 5 Department of Geography, King’s College London, London, United Kingdom; 6 Centre for Climate Adaptation & Environment Research (CAER), University of Bath, Bath, United Kingdom; 7 Scientific Consulting Group, Gaithersburg, MD, United States of America; 8 HKV consultants, Delft, The Netherlands; 9 Center for Climate Change and Sustainability Studies, University of Ghana, Accra, Ghana; 10 School of Public Health, Yale University, New Haven, CT, United States of America; 11 International Institute for Environment and Development (IIED), London, United Kingdom; 12 Department of Civil and Environmental Engineering, University of Liverpool, Liverpool, United Kingdom; 13 Invisible Flock Studio, Leeds, United Kingdom; 14 Faculty of Built and Natural Environment, Tamale Technical University, Tamale, Ghana

**Keywords:** climate change adaptation, coproduction, locally led adaptation, climate justice, adaptation assessments, flood risk modelling

## Abstract

Systematic assessments of climate change adaptation are critical for monitoring progress and planning effectively, but current approaches are limited in their scope, accuracy, and relevance to local contexts. Here, we present an improved approach using coproduction to quantitively assess adaptation based on local knowledge and priorities. This is applied to locally led adaptation (LLA) to flood risk in Tamale, Ghana, to provide the first quantitative assessments of this increasingly common adaptation practice. Through a multi-year process, including community marble distribution, focus groups, and household surveys, 11 LLA solutions were assessed. Assessments were based on adaptation success criteria that mattered most to local communities and included important considerations that are commonly missing from technical assessments, including multiple risk-reduction mechanisms, equity, sustainability, and co-impacts. Community-based and behavioural LLA solutions, such as collective action and tree planting, were deemed most effective, whilst structural and technical solutions were ranked lower. By integrating these assessments into a flood risk model, we show that LLA approaches significantly reduced flood risk overall but did not address existing inequalities. Our results showcase the potential of coproduction to increase the scope and robustness of adaptation assessments and highlight practical challenges of delivering on the LLA principles in real-world settings.

## 1. Introduction

Adaptation is critical for mitigating the impacts of climate change, such as increased flood hazards [1, 2]. Effective, equitable, and resilient adaptation requires rigorous planning, informed by reliable scientific evidence and local and Indigenous knowledge, and with meaningful participation of citizens and communities [3]. Central to this process is an accurate understanding of the effectiveness (i.e. ability to achieve a predefined goal, such as reducing flood risk) of adaptation solutions in different contexts, for example by representing adaptation in risk models used for climate research, policy, and planning [4, 5].

However, accurately assessing adaptation actions remains a challenge [6–9]. Quantitative assessments remain primarily limited to specific solutions, stakeholders, scales, and success criteria. Behavioural adaptation is not typically covered, despite being the most common solution [10]. People with direct experience are not typically engaged, despite being the most impacted and possessing highly relevant knowledge [5, 6]. City and neighbourhood scale assessments are most common, despite much adaptation occurring hyper-locally (e.g. at the household level) [11, 12]. Assessments focus heavily on hazard and exposure, despite recognition that vulnerability, adaptive capacity, and equity are critical [11–15]. Neglecting these considerations results in inaccurate assessments and contributes to maladaptation, leading to increased climate risk and inequalities [7, 16, 17].

A promising approach for improved assessments is the coproduction of knowledge, whereby academics and non-academic partners work together using transdisciplinary approaches to prioritise, research, and deliver actionable knowledge [18–21]. However, whilst coproduction has been applied to climate research, policy, and services, it is yet to be applied to adaptation assessments [22, 23]. Coproduction could improve the accuracy, scope, and relevance of assessments by enabling meaningful participation of local actors to determine success criteria and leverage multiple epistemologies, including local and Indigenous knowledge [24]. This basis could integrate context-specific considerations of multiple risk-reduction mechanisms (e.g. exposure and vulnerability reduction), sustainability (e.g. effectiveness over time), co-benefits and trade-offs, and inequalities (e.g. gender), whilst also empowering communities to take the lead on adaptation action [21, 22].

The aim of this paper is to advance and test the coproduction of adaptation assessments and their use in quantitative risk models. We develop this novel approach and apply it to assess locally led adaptation (LLA) and evaluate quantitatively its effects on risk and equality [25]. LLA refers to solutions led by local communities empowered to self-determine their objectives and strategies of adaptation [26, 27]. This is favoured, especially in low- and middle-income countries, because it can be based on local knowledge and tailored to local conditions, thereby leading to more effective, equitable, and legitimate outcomes at lower cost and greater speed [28, 29]. LLA commonly manifests as non-structural solutions at the community or household level (e.g. behavioural changes and collective action) that cannot be assessed quantitatively using existing methods [10]. Therefore, there is a paucity of LLA assessments which results in limited understanding of its ability to equitably mitigate climate risks, undermining its inclusion in planning and financing and limiting widescale deployment [30, 31].

## 2. Methods

### 2.1. Study location

Tamale, Ghana, faces challenges associated with a rapidly growing population, inadequate public services, and climate change [32]. The population has tripled in 25 years to ∼750 000, driving rapid urbanisation and expansion [33]. Many residents are highly vulnerable to climate hazards, with 21% living in multidimensional poverty [33]. Climate hazards, including flooding, are increasing in frequency and magnitude, now occurring annually [34–36]. Climate change adaptation is constrained by limited human and financial resources and governance challenges [35, 37]. Such challenges are common to secondary cities in Africa, and therefore Tamale provides a suitable case study [38].

### 2.2. Coproduction approach

This research is part of a wider coproduction process that began in 2022 (described in detail elsewhere [39, 40]) following the ‘loops and building blocks’ model [41]. A core coproduction team of >50 academics, governmental and NGO officials, traditional leaders (i.e. chiefs), and community representatives led the research, which has directly involved >1000 participants. The research focus and questions were co-defined during a three-day workshop, in which pluvial flooding was selected because it was deemed to affect the largest proportion of Tamale residents. Following a coproduction phase focussing on governance [35], LLA emerged as an important tool, but questions remained around its effectiveness and equity [40]. LLA solutions (table 1) were identified by the coproduction team in workshops and community walk throughs, including solutions that were currently being practiced and that are driven by community members (i.e. locally led) and primarily occur on the household level. To some extent the framing of LLA was applied in retrospect, with some solutions occurring autonomously (i.e. without community planning) and without risk information. Therefore, not all of the LLA principles were followed from the outset, although we argue such pragmatic application of LLA is useful and necessary [28]. Three flood exposed communities (Kalariga, Nalung, and Koblimahagu) were selected for study to represent a range of socioeconomic statuses.

**Table 1.** Common LLA solutions to flooding in Tamale, Ghana, as identified by the coproduction team. Individuals pictured provided verbal consent for their image to be used. Pictures were all captured in Tamale by the coproduction team.

Adaptation solution	Description	Picture example
Community practice	Collective action to improve community spaces, e.g. drain clearing, waste disposal, or erosion management.	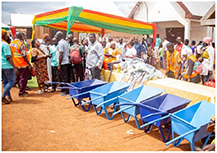
Planting trees	Planting and/or managing plants (e.g. trees or grasses) to reduce flood generation or velocity.	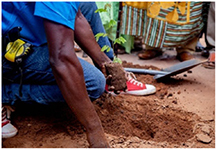
Protecting valuables	Storing valuables (e.g. money, documents, electronics) in flood-proof locations, e.g. elevated or watertight containers.	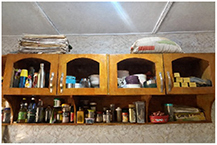
Community planning	Coming together to organise or plan flood risk reduction activities, e.g. lobbying local governments or planning evacuation.	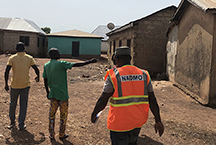
Flood education	Engaging with awareness and education activities about flood risk and adaptation, e.g. in organised workshops or online.	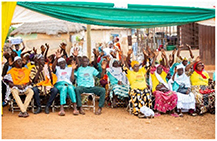
Walls and embankments	Erecting barriers between the house and the direction of flood water, e.g. sandbags or compound wall.	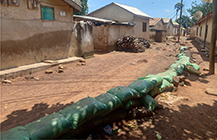
Structural supports	Housing modifications to improve strength of the walls or roof of the house, e.g. block pillars or piers.	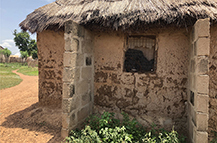
Emergency provisions	Storing essential items like food, water or money to help survive and/or recover from flooding.	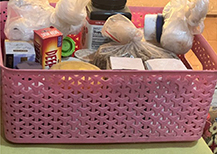
Raised elevation	Building the house on a platform to raise it above the typical flood water level.	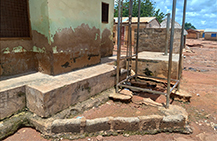
Early warning systems	Using early warning systems to inform decisions, e.g. to evacuate.	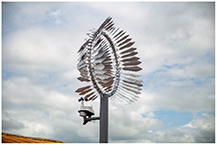
Household drainage	Household level conduits that move water out of or away from the house.	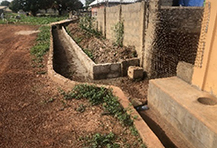

**Figure 1.**
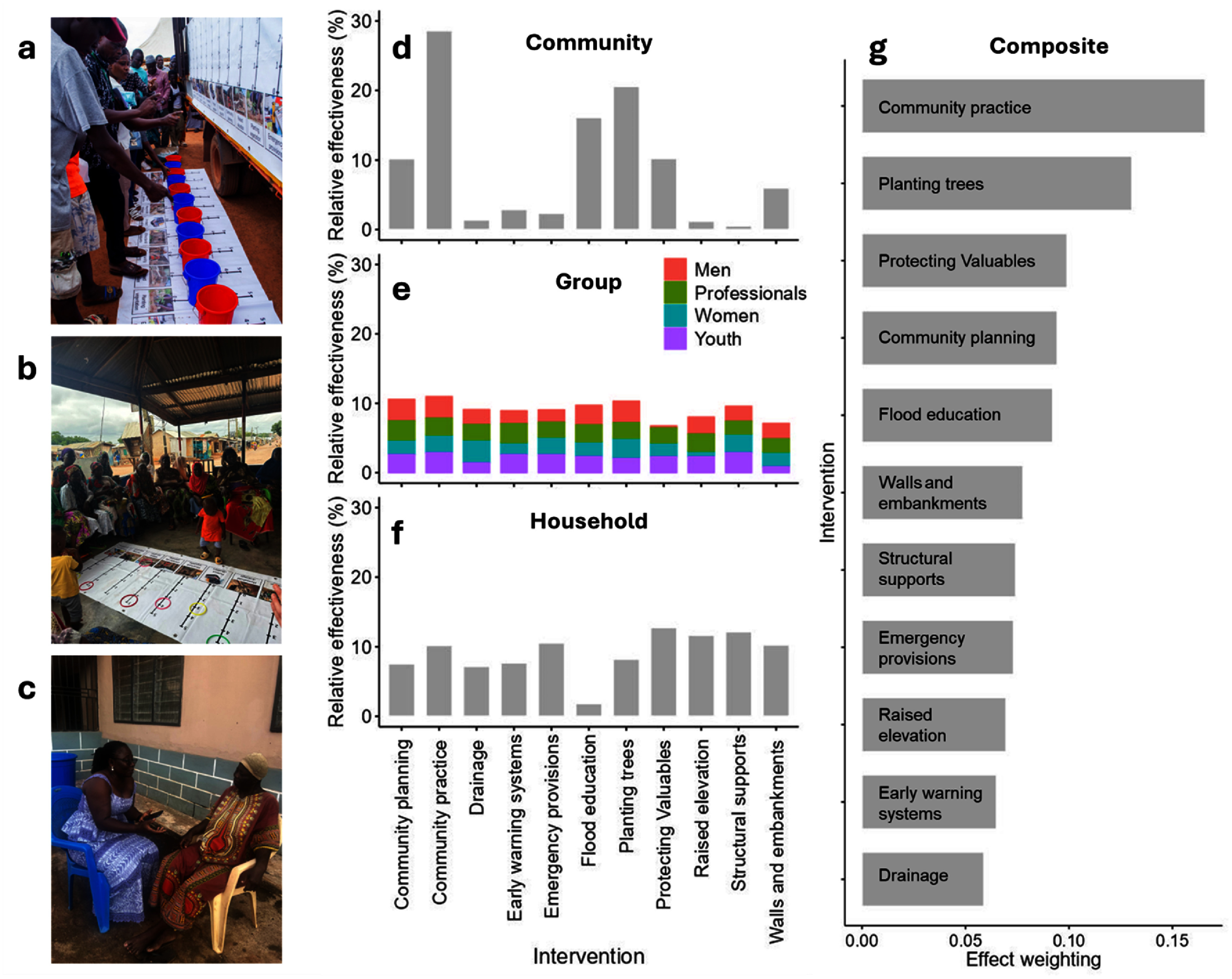
Three participatory activities were used to assess adaptation solutions: (a) community marble distribution, (b) demographic focus groups, (c) household survey of three communities (Nalung, Koblimahagu, and Kalariga) (credit: Cynthia Awuni). The relative importance of adaptation solutions was calculated based on (d) community marble distribution, (e) demographic focus groups, and (f) household practice rates, and combined in (g) a composite assessment indicator normalised to one.

Three participatory activities were used: community marble distribution (*n* = 205 people) (figure 1(a)), demographic (females, males, youth, professionals) focus group scoring (*n* = 8 groups of 15–30 people) (figure 1(b)), and household practice rates (*n* = 285 households) (figure 1(c)). All activities were explained by a member of the coproduction team from the national disaster management organisation (NADMO) who spoke both English and Dagbani (the local language). Effectiveness was not defined by the research team, leaving participants to determine criteria themselves.

Community marble distribution (i.e. the bean method), used to overcome literacy barriers [42], took place in Nalung in June 2024. ∼400 attendees from flood exposed communities were invited to participate by distributing 11 marbles amongst 11 buckets, each associated with an adaptation intervention, relating to the effectiveness of that intervention in reducing flood risk (figure 1(a)).

**Figure 2.**
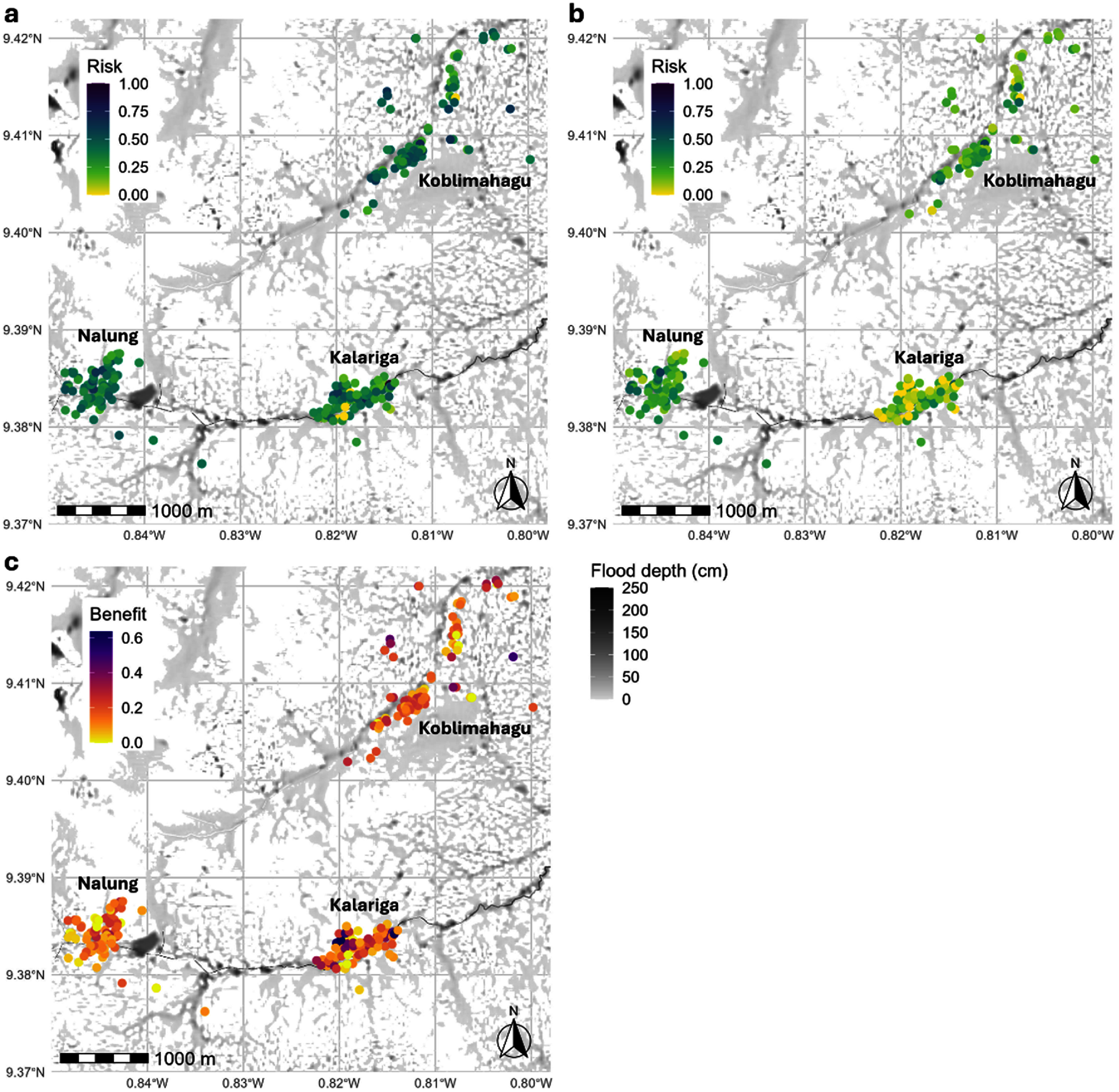
(a) baseline flood risk, (b) adapted flood risk, and (c) benefit of adaptation for households in Tamale. Flood depth represents the modelled depth during a 10 year return period event.

Eight focus group activities were conducted in May 2024 in communities, including 15–30 people and lasting 1–2 h. To ensure a diversity of perspectives, for example those of typically marginalised groups, focus groups consisted of females, youths (ages 12–17, mixed-sex groups), and males, who were recruited by community representatives, as well as staff from four NADMO local offices. For each intervention a different participant led a discussion about its effectiveness at reducing flood risk. Participants ranked the importance of an intervention 0–5 by placing a hoop over the score, which was recorded by researchers (figure 2(a)). Participants were encouraged to consider the relative effectiveness of interventions (i.e. not to rank everything 5). Notes from focus groups are in the supporting information (SI). Results were normalised by the total score of each group and by the number of groups. We give equal weighting to each group because the process was designed to capture diverse lived experiences of flood risk rather than to privilege particular forms of expertise.

The design and deployment of the household level questionnaires have been described in detail elsewhere [40]. 301 surveys were conducted in October 2023 (figure 1(c)), representing 5%–10% of the households in each community. Surveyors aimed to recruit one participant (typically the household head) from every tenth house; if a household could not be recruited, surveyors moved onto the next household. Data was recorded in English using Kobo toolbox [43] but all surveyors were fluent speakers in Dagbani.16 households were removed because questions had been omitted or the location had not been recorded, leaving 285 questionnaires for analysis. Here, only responses directly relating to the practice of interventions, vulnerability, and exposure were used (SI). The number of households practicing each intervention was summed and normalised by the total number of adaptation practices reported across all households to provide a proportional representation of each intervention’s relative prominence.

### 2.3. Risk estimates

Risk (i.e. the potential for adverse consequences) is conceptualised as a composite of hazard, exposure, and vulnerability [44, 45]. Hazard and exposure refer to the probability of physical exposure to a specific hazard [45].

\begin{align*}{\mathrm{Hazard} \ \&amp; \,\, \mathrm{Exposure}} = { }\sum\limits_{i \in \left\{ {1,5,10} \right\}} {P_i}{D_i}\end{align*}

where *i* is the return period, *P* is the probability of such an event occurring in any given year, and *D* is the depth of flooding

*D* is a combination of self-reported and modelled depths, prioritising self-reported due to model limitations discussed below and to respect our community-centred approach. For example, if a household reports to not be flooded at a specific *i,* we assume *D*= 0, even if the hydraulic model reported a higher *D*. More commonly (*i_1_* = 214, *i_5_* = 204, *i_10_* = 213), a household reported *D*> 0 when the hydraulic model did not, in which case, for each specific *i*, the median modelled *D* across households in the relevant community with modelled depths >5 cm was used. The median was selected as a robust, conservative estimate of typical community *D,* which is less sensitive to extremes than other substitutions (e.g. mean or percentiles) and therefore suitable given the relatively small number of households with modelled *D* > 5 cm in each community, including some with locally high *D*, e.g., near drainage channels. This is intended as a pragmatic proxy of exposure, which suits our model’s aim of assessing LLA effectiveness during a ‘typical’ flood event, i.e. a frequently occurring flood of the extent and depth participants were asked to consider.

Vulnerability refers to the susceptibility of an exposed population to be negatively impacted by a hazard [46]. We used a rank composite social vulnerability index relevant for sub-Saharan Africa, including demographic, economic, social sub-indices [47–50]. For each sub-index, households are ranked by each indicator and then averaged. The demographic sub-index is comprised of indicators related to household size and education level [48, 51]. The economic sub-index incorporates diversified sources of income, measured using Shannon’s entropy index, and climate sensitive occupations, defined based on self-reported impacts on household economic activities from previous flooding events [48, 52–54]. The social sub-index combines indicators on access to climate change information (based on access to early warning systems, flooding education, and community practice and planning) and dependence on natural resources [48, 51, 54].

Quantitative adaptation assessments (figure 1(g)) were calculated by summing the results of the three participatory activities for each intervention and normalizing to one. We give equal weighting to each activity because each captures a different aspect of community judgment: marble ranking reflects collective prioritisation, focus groups allow discussion amongst demographic groups, and household surveys capture individual responses. By combining them equally we aim to avoid prioritising the experiences of one group. Adaptation scores for each household were calculated by summing the effect weightings of each intervention practiced. To ensure equal contribution of the dimensions to baseline risk, hazard and exposure and vulnerability were normalised between 0 and 1 using min-max scaling, and to ensure a normal distribution hazard and exposure was additionally normalised using a square root transformation. Baseline flood risk was described by the widely applied equation (2).

\begin{align*} &amp;{\text{Baseline Flood Risk}} \nonumber\\ &amp; \quad = {\mathrm{Hazard} \ \ \mathrm{Exposure} \times \mathrm{Vulnerability}}.\end{align*}

Adaptation was not normalised to maintain the effect weightings determined in the coproduction process. Adapted flood risk was estimated using equation (3).

\begin{align*} &amp;{\text{Adapted Flood Risk}} \nonumber\\ &amp; \quad = {\text{Baseline Flood Risk}} \times \left( {1 - {\mathrm{Adaptation}}} \right){ }\end{align*}

where adaptation refers to the adaptation score calculated for each household, as described previously. This is a commonly applied risk equation (e.g. [44, 45]) whereby adaptation is treated as a modifier of total risk, rather than modifying hazard and exposure or vulnerability separately, which is suitable here because we do not aim to explicitly model different risk reduction mechanisms.

Adaptation benefit (i.e. the reduction in risk achieved due to adaptation) is calculated using equation (4).

\begin{align*} &amp;{\text{Adaptation Benefit}} \nonumber\\ &amp; \quad = {\text{Baseline Flood Risk}} - {\text{Adapted Flood Risk}}.\end{align*}

### 2.4. Hydraulic modelling

We set up a 1D/2D coupled hydraulic model to estimate flood depth and extent during different precipitation events, using HEC-RAS version 6.4.1 [55]. We modelled an area of 46 km^2^, inclusive of the three target communities. A basis DTM from airbus WorldDEM was used with a 12 m resolution. Main drainage channels were digitised based on satellite imagery and field visits and implemented in 1D. Ten bridges or culverts were implemented along primary drainage channels. Land use was estimated from the European Space Agency WorldCover (2020) maps at 10 m resolution [56],and used to assigned Manning’s roughness values based on the Ven Te Chow 1959 handbook and infiltration based on the soil conservation service curve number method [57, 58]. Inundation depths <5 cm were excluded to avoid including model artefacts. We modelled 1-, 5-, and 10 year return period, 6 h precipitation events to which Tamale is vulnerable. Including this relatively small range of flood scenarios is appropriate to provide aggregate estimates of hazard and exposure but it does limit the relevance of assessments to smaller flood events. We generated hyetographs from intensity duration frequency curves derived in 1974 because suitable recent data was not available. Comparison with recent estimates of daily precipitation in Tamale shows reasonable agreement (15.4%–19.6% difference) and is in line with those of sub-daily events in other regions in Ghana [59]. However, the intensity of precipitation in the short-duration events we consider is likely to have increased since 1974, likely introducing negative bias in our hazard estimates and posing a limitation to the assessment of LLA. Method described in detail in SI.

### 2.5. Statistical analysis

For each community, an independent *t*-test was conducted to compare baseline and adapted risk. Effect sizes were calculated using Cohen’s *d*, and group means, mean differences, and associated *p*-values were extracted for each community. A two-sample Kolmogorov–Smirnov test was used to compare baseline and adapted risk distributions, and Cohen’s d was computed by decile to quantify adaptation effects across the risk spectrum. Risk inequality was evaluated using Lorenz curves and Gini coefficients for both scenarios. K-means clustering (*k* = 3) was applied to standardised baseline risk, adapted risk, and adaptation benefit variables to identify risk–benefit groups. Cluster differences in hazard exposure, vulnerability, and adaptation score were examined using two-way and one-way ANOVA with Tukey post hoc tests to assess pairwise differences among clusters. Analyses conducted in *R* version 4.3.1.

## 3. Results

### 3.1. Adaptation assessments

Participants considered the effectiveness of solutions during different magnitude flood events and the risk reduction mechanism, e.g. preventing water from entering the household (i.e. reduced exposure), preparing to better cope with flood events (i.e. reduced vulnerability), or building capacity for future responses (i.e. increased adaptive capacity). Discussion was based on experience with solutions (i.e. tried and tested), revealing a variety of experiences and centring assessments on equity, e.g. protecting valuables is pointless if you have no valuables (evidenced in focus group notes in the SI for women’s group [WG]1, WG2, youth group [YG]1, YG2, men’s group [MG]1, and NADMO group [NG]2). Generally, solutions that require maintenance deemed too intensive, costly, or unachievable (e.g. hindered by complex governance) were considered unsustainable, and therefore evaluated less favourably, e.g. keeping drainage clear of solid waste (community practice) or maintaining trees (WG1, WG2, MG1, NG1, NG2). Avoiding unintended consequences (i.e. trade-offs) was prioritised in assessments, especially if solutions exacerbated risk for neighbours, e.g. offsetting flood waters by building houses on platforms (WG1, YG1, YG2, MG1). The co-benefits of some solutions were considered important, especially relating to community building, e.g. community planning (WG1, WG2, YG2, MG2, NG2).

A variety of solutions were deemed effective, with preference for community-based and non-technical approaches (figure 1(g)) which accounted for four of the top five solutions. Structural and technical solutions (e.g. raised elevation and early warning systems) were found to be relatively ineffective.

Participatory activities yielded different assessment outcomes. The community activity revealed the largest range in effectiveness, from 0.5% for household structural supports to 28.6% for community practice (figure 1(d)); conversely, the group activity yielded the smallest range, with all solutions between 6.3% and 11.2% (figure 1(e), figure 1 in SI). Community assessments disproportionately valued community-based solutions that increase adaptive capacity, potentially reflecting social pressure [60]. The small differences between solutions in the group assessments is likely driven by moderation effects classically observed in group decision making, whereby groups compromise to reach consensus [61]. However, amalgamating groups can mask differences within them, e.g. male participants ranked protecting valuables lower than other groups. The household practice rates assessment (figure 1(f)) effectively tested ‘do people practice what they preach?’. The answer is no. Community-based solutions were practiced less than would be assumed based on their assessments otherwise, and structural solutions were practiced more. Whilst practice rates are indicative of perceived effectiveness, there are other reasons why solutions may or may not be practiced [40].

### 3.2. Effects of locally led adaptation on risk distribution

**Figure 3.**
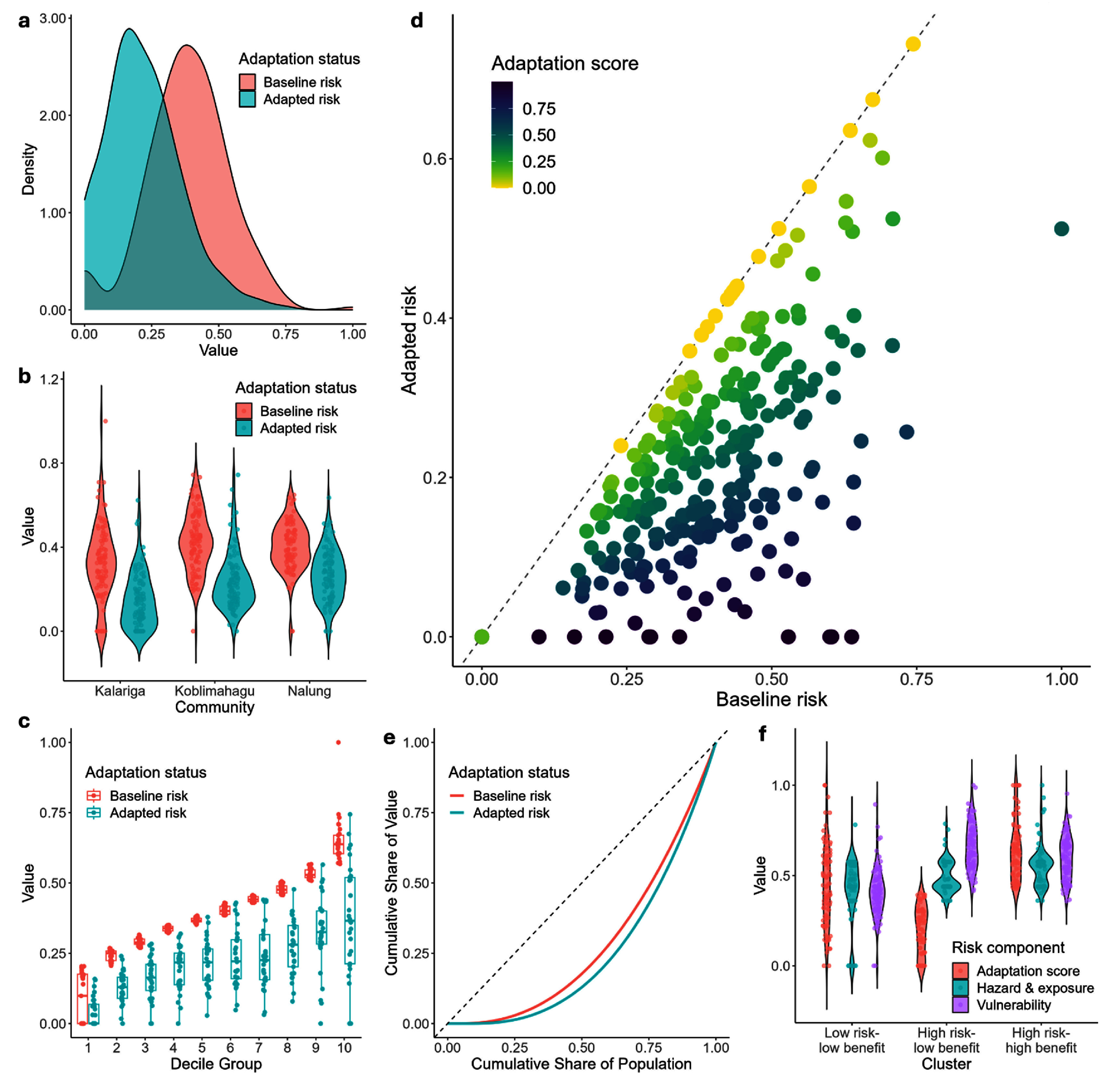
(a) Kernel density plots showing baseline and adapted risk for the whole sampled population. (b) Violin plots showing baseline and adapted risk for each community. (c) Box plots of baseline and adapted risk for each decile of baseline risk. (d) Scatter plot of baseline risk ranking and adapted risk ranking with colour representing adaptation benefit and the grey dashed line representing the identify line. (e) Lorenz curve of baseline and adapted risk and the grey dashed line representing the line of equality. (f) Violin plots showing risk components distributions of three clusters identified using principal component analysis.

High baseline flood risk was widespread, but every community also included households of low risk (figure 2(a)). The inclusion of LLA significantly reduced risk in every community (*P* < 0.001), with the largest reduction observed in Koblimahagu (mean difference = 0.185, Cohen’s *d* = 1.33), followed by Kalariga (mean difference = 0.178, Cohen’s *d* = 1.11) and Nalung (mean difference = 0.136, Cohen’s *d* = 1.14). When adaptation is included, most low-risk households are in Kalariga (figure 2(b), figure 3(b))—the community with the perceived highest socioeconomic status—which also includes the most households with large adaptation benefit (figure 2(c)). Households of high and low benefit exist in proximity, reflecting baseline risk patterns and widely varying adaptation scores (figure 2(c)).

Including all LLA solutions reduced overall risk by 57.6%, representing a significant decrease (Kolmogorov–Smirnov, *D* = 0.50519, *P* < 0.05) from baseline risk (mean = 0.383, median = 0.384, sd = 0.155) to adapted risk (mean = 0.217, median = 0.203, sd = 0.140) (figure 3(a)). Adaptation reduced risk significantly for every decile (*P*< 0.05); central deciles are similarly reduced (Cohen’s d mean deciles 2–8 = 2.49, ±0.21), with exceptions at the lowest (Cohen’s *d* = 0.7) and highest (Cohen’s *d* = 1.82) deciles. A gradient in risk reduction is observed across deciles, with the mean difference between baseline and adapted risk greatest in the tenth decile (0.28 ± 0.19) and progressively decreasing to the first decile (0.05 ± 0.06), indicating a greater effect of adaptation for higher-risk households. However, some high-risk households did not benefit at all (i.e. yellow points on the identify line in figure 3(c)). This is also reflected by the Lorenz curve (figure 3(e)) and Gini coefficients which evidence an increase from low risk inequality in the baseline scenario (Gini = 0.222 16) to medium risk inequality in the adapted scenario (Gini = 0.357 32) [62].

Distinct groups of households were characterised by significant differences in the components of risk (hazard and exposure, vulnerability, and adaptation score) (figure 3(f)). The biggest difference is the significantly lower adaptation score (i.e. fewer and/or less effective solutions practiced) in the *high risk—low benefit* group compared to *high risk—high benefit* (*p* < 0.0001), suggesting this is the primary driver for the disparity between the groups. Whilst hazard and exposure were similar, a higher vulnerability (*p* < 0.0001) in the *high risk—low benefit* group suggests that higher vulnerability is associated with lower adaptation score [40]. The *low risk–low benefit* group has significantly lower hazard and exposure (*p* < 0.001) and vulnerability (*p* < 0.0001) (i.e. risk drivers) than the other groups, but a large range of adaptation scores, including some of the highest. Hence, a lower baseline risk provides decreased potential for risk reduction overall, via a ‘diminishing returns’ effect [63].

## 4. Discussion

### 4.1. Coproduction approaches to adaptation assessments

Coproduction enabled the quantitative assessment of a wide range of adaptation solutions, including behavioural, hyper-local, and context-specific solutions. Behavioural changes are the most common adaptation solutions globally, motivating transformative change and protecting against severe hazards [10]. Hyper-local solutions can be scaled rapidly to reduce short-term risks and catalyse wider system-level adaptation [27, 28, 30]. Adaptation outcomes are determined by appropriateness to context [6, 7, 9]. Coproduction enables robust assessments of these strategies, and a wider range of possible solutions, supporting their inclusion in climate risk models, policy, and funding.

The best ways to assess adaptation are disputed [7]. Given the range of objectives, adaptation should be assessed against criteria determined by people who are directly impacted [24, 64]. Here, coproduction enabled holistic assessments based implicitly on criteria determined by beneficiaries, who can assess adaptation with accuracy, as measured through their consistent assessment of interventions before and after a flood [65]. The criteria selected are important in most contexts but are often overlooked. For example, assessments raised considerations of gender and age bias, which are a primary control of adaptive capacity, and showed that different groups assess LLA solutions differently (figure 1(e), figure 1 in SI) [66]. Equity considerations were central, the neglect of which contributes to increasing adaptation gaps [67]. A range of risk reduction mechanisms were considered that makeup optimal adaptation pathways [68]. Consideration of co-benefits and trade-offs demonstrates understating of the interlinked effects common to adaptation practice [27]. Prioritizing sustainability is important because many adaptation projects that are initially successful fail due to maintenance challenges [67, 69].

Coproduced assessments are both context-relevant and context-dependent. Our process leveraged subjective adaptation assessments, which can give legitimacy to diverse perspectives but complicates comparison across and translation to different contexts [69]. Furthermore, subjective assessments are vulnerable to bias; several social bias effects were observed, e.g. peer pressure [60], group moderation effects [61], and inconsistency between people’s words and their actions. Perceived risks and benefits can differ from actual and may be less relevant in a climate-changed future (e.g. more severe floods), potentially compromising their accuracy. Basis on past experiences largely limits assessments to solutions that are familiar, thereby reducing potential to introduce new solutions which could facilitate transformative change. For example, non-technical solutions, which are widely practiced (e.g. protecting valuables), were preferred despite representing coping or incremental adaptation [70]. Technical solutions (e.g. early warning systems) are relatively uncommon in Tamale and were not prioritised, despite potential to protect communities during severe floods [71].

Coproduction and adaptation processes cannot be directly transferred into new contexts; however, the coproduction and analysis framework outlined here is sufficiently flexible to be adapted for diverse community-specific needs, e.g. use of technology, local capacity, and language requirements [27]. A major challenge remains in up-scaling coproduced knowledge beyond those directly involved and in translating outcomes to actionable evidence [72]. By novel integration of coproduced assessments into a flood risk model, we quantify effects of LLA on risk and equity and deliver evidence and tools that can be used in adaptation planning.

### 4.2. Effects of locally led adaptation

We found that whilst LLA approaches reduced flood risk in Tamale, they did not address existing inequalities within and between communities. Some of the highest risk households benefited the least, leading to widening adaptation gaps [73, 74]. Vulnerability was associated with lower levels of adaptation which led to a large group of high-risk households receiving low adaptation benefit. We attribute this apparent failure of LLA to address inequalities to the retrospective application of the LLA framing, which meant that some LLA principles (e.g. addressing structural inequalities) were poorly followed [28]. This highlights the importance of adhering to the LLA principles at all stages of the adaptation process. In this context, LLA would benefit from top-down facilitation to increase the enabling environment, for example by helping to develop climate risk information and address barriers to adaptation.

In Tamale, a range of LLA solutions were considered effective (figures 1(d)–(f)), suggesting participants valued diverse adaptation pathways. Not only does this indicate that local knowledge corresponds to scientific knowledge in this area, but it further highlights the potential of LLA approaches, which are typically holistic, non-technical, and small scale (figure 1(g)) [10, 27, 68]. These solutions were deemed most effective here and elsewhere [75]. In contrast, structural and technical solutions were deemed ineffective, potentially reflecting unrealistic expectations (e.g. total risk reduction) or challenges (e.g. limited local capacity) in their proper implementation [76, 77].

Due to the pseudo-quantitative nature of our model, the overall risk reduction of 57.6% we report reflects a relative effectiveness. This is in common with flood risk modelling frameworks and is still useful to compare LLA solutions and investigate equity [44]. Validating flood risk and adaptation models is a crucial but challenging step [78]. Whilst we did not actively validate the outcomes of our model, our framework includes an element of validation (i.e. because assessments are based on real experiences); however, *ex post* validation with participants following flood events would be useful.

## 5. Conclusion

We demonstrate the coproduction of quantitative assessments of climate change adaptation. With basis in local knowledge, values, and lived experiences, coproduced assessments reflect a diversity of success criteria that matter most to communities, and that are commonly missing from technical assessments. The holistic approach allows for assessment of a broader range of solutions, particularly non-structural and community-based measures that are often overlooked. This is a significant methodological advancement which could improve the accuracy, actionability, and scope of adaptation assessments.

Our findings highlight both the potential and the challenges of LLA. Whilst in this case LLA solutions significantly reduced overall risk, they did so unevenly, often excluding the highest-risk households. LLA approaches must be central to building resilience in communities, but they must be complimented by top-down facilitation, ensuring that resources, technical support, and capacity development are directed to the most vulnerable.
